# A database of atmospheric nitrogen concentration and deposition from the nationwide monitoring network in China

**DOI:** 10.1038/s41597-019-0061-2

**Published:** 2019-05-09

**Authors:** Wen Xu, Lin Zhang, Xuejun Liu

**Affiliations:** 1 College of Resources and Environmental Sciences; National Academy of Agriculture Green Development, Key Laboratory of Plant-Soil Interactions of MOE, China Agricultural University, Beijing, 100193 China; 2 Laboratory for Climate and Ocean-Atmosphere Sciences, Department of Atmospheric and Oceanic Sciences, School of Physics, Peking University, Beijing, 100871 China

**Keywords:** Atmospheric chemistry, Environmental monitoring

## Abstract

Atmospheric nitrogen (N) deposition has increased substantially across China since 1980; however, data for N deposition fluxes since the 2000s has been very limited. Understanding and mitigating the impacts of N deposition requires long-term quantification of dry as well as wet deposition of key reactive nitrogen (Nr) species. Here we present a dataset for inorganic N concentrations and deposition for the period 2010–2015 in China, compiled from the nationwide deposition monitoring network. The dataset comprises information from 32 monitoring sites on concentrations and bulk deposition (wet plus part of dry deposition) fluxes of NH_4_^+^-N and NO_3_^−^-N, air concentrations and dry deposition fluxes of the major Nr species NH_3_, NO_2_, HNO_3_, and particulate NH_4_^+^ and NO_3_^−^. This unique database is available inter alia to advance understanding of the spatial patterns of inorganic N concentrations and deposition in China and its associated effects, constrain primary Nr (e.g., NH_3_, NO_*x*_) emission inventories, and validate outputs of atmospheric chemistry and transport models.

## Abstract (summary)

**Table:** 

Design Type(s)	observational design • data integration objective
Measurement Type(s)	nitrogen compounds
Technology Type(s)	environmental monitoring
Factor Type(s)	temporal_interval • geographic location
Sample Characteristic(s)	China • city • rural area • coast • grassland area • forested area

Machine-accessible metadata file describing the reported data (ISA-Tab format)

## Background & Summary

The deposition of reactive nitrogen (N) from the atmosphere to the surface is an important component of the human-accelerated global N cycle and a serious form of atmospheric pollution. Reactive nitrogen (Nr) comprises both oxidized (e.g. NO, NO_2_, HNO_3_) and reduced (NH_3_) gases and their particle-phase nitrate (NO_3_^−^) and ammonium (NH_4_^+^) equivalents. Excess Nr deposition results in adverse ecological effects, including the loss of biological diversity^1^, nutrient imbalance^2^, soil acidification^3^, and eutrophication of water bodies^4^. Nitrogen deposition of oxidized N has decreased or stabilized in Europe and the U.S. since 1990, mainly due to reductions in emissions of NO_*x*_ (NO + NO_2_)^5–7^. In contrast, growing agricultural and industrial activities in China have led to increasing emissions of both NH_3_ and NO_*x*_ since the 1980s^8^. Although the trend has slowed recently with the introduction of strict air pollution measures, the atmospheric Nr deposition in China consequent on the large emissions of Nr to the atmosphere is of widespread concern^9^.

Atmospheric deposition occurs via wet and dry deposition pathways. Wet deposition refers to removal of gases and particles from the atmosphere by precipitation events (rain and/or snow), whilst dry deposition is the transport of gases and particles to surface via turbulent exchange and gravitational settling in the absence of precipitation. Compared with wet deposition, data for dry deposition fluxes were much sparser in China and other countries worldwide^10^. This is because wet deposition can be directly quantified from chemical analysis of collected bulk precipitation samples, whereas direct measurement of dry deposition is technically challenging and needs to include a wide range of N-containing compounds in both gaseous and particle phases^11^. The direct measurement of dry deposition fluxes via micrometeorological methods requires complex and expensive instruments^12^ and therefore cannot be applied over large domains or for long time series. Instead, the inferential method can be used for estimating dry deposition at large spatial and long-term scales, despite the associated uncertainties^10,13^. In this approach, dry deposition fluxes are estimated as the product of the atmospheric concentrations of the species of interest and their deposition velocities, the latter of which are inferred from meteorological variables and land-use type^13^.

National measurements of nitrogen deposition did not exist in China until the instigation in 2004 of the Nationwide Nitrogen Deposition Monitoring Network (NNDMN) operated by China Agricultural University^14^. Initially this network comprised only measurements of bulk (wet) N deposition. In 2010, simultaneous measurements of air concentrations and associated dry deposition fluxes of five major N_r_ species (i.e., gaseous NH_3_, NO_2_ and HNO_3_, and particulate NH_4_^+^ and NO_3_^−^), were added^11^. Examination of the more comprehensive data between 2010 and 2015 has shown both high Nr concentrations and deposition fluxes and a high degree of spatial variability across China^9,11,15^, for example greater Nr pollution in the northern region than in the southern region, especially in rural areas^9^. Analysis has also revealed the equal importance of dry and wet deposition at a national scale^11^, which demonstrates the need to include both dry and wet/bulk deposition in evaluating the effects of N deposition on eco-environmental health. In summary, despite achievement of effective controls of SO_2_ and NO_x_ emissions, the absence of regulation or legislation regarding agricultural NH_3_ means that China has still been subject to high levels of N_r_ deposition in recent years^9,11,15^.

**Fig. 1:**
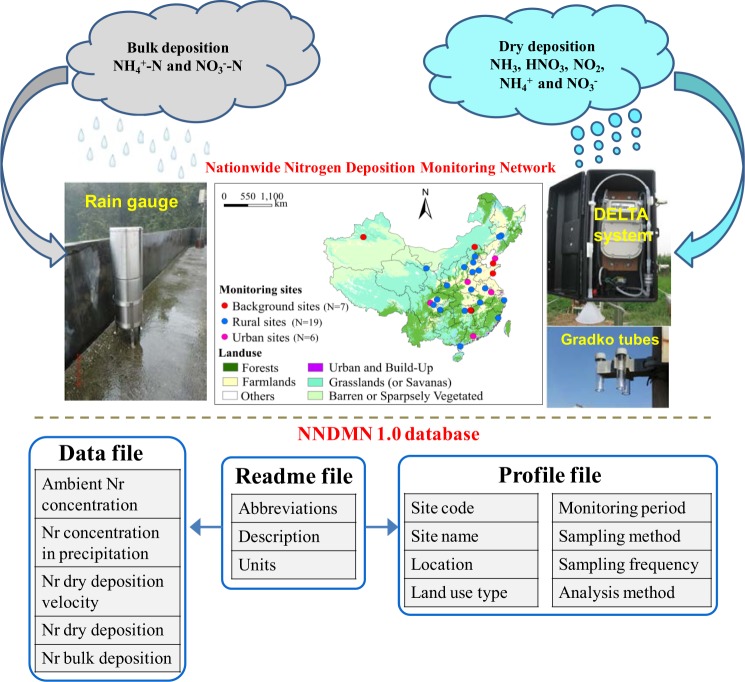
Overview of distribution of 32 dry and bulk deposition monitoring sites and their respective instruments in the nationwide nitrogen deposition monitoring network (NNDMN) in China and the framework of the NNDMN 1.0 database.

The World Meteorological Organization (WMO) Global Atmosphere Watch programme (GAW) recently completed an assessment of global N wet and dry deposition, but only a few Chinese monitoring sites were included for validation of modeled wet deposition, and none for validation of dry deposition^10^. The absence of an open-access database for atmospheric Nr composition and deposition in China, especially dry deposition, strongly motivates us to provide global scientists with the dataset of Nr deposition from the NNDMN. We define this dataset as NNDMN 1.0 version, and it comprises three files, the ‘data file’, a ‘profile file’ and a ‘readme file’ (Fig. 1). The dataset contains monthly average air concentrations and dry deposition fluxes of NH_3_, NO_2_ and HNO_3_, and particulate NH_4_^+^ and NO_3_^−^, and volume-weighted mean concentrations and bulk deposition fluxes of NH_4_^+^-N and NO_3_^−^-N, as well as detailed information on each site including longitude, latitude and land use, and the methods of field sampling and laboratory analysis. This database will enable scientists and policymakers to explore spatiotemporal trends of N deposition in China, validate modelling results and assess the ecological burden of N deposition on sensitive ecosystems (e.g., forest and grassland) in China. At present most sites have about 5 years of measurements and the intention is to add ongoing data collection from the NNDMN to this open-access dataset to enable longer-term trends in bulk and dry N deposition in China to be followed.

## Methods

### Database structure

The NNDMN 1.0 database consists of three files (Fig. 1). The ‘data file’ provides monthly mean data on the concentrations of NH_4_^+^-N and NO_3_^−^-N in precipitation, bulk N deposition fluxes of NH_4_^+^-N and NO_3_^−^-N, monthly integrated precipitation amount, and the concentrations and dry deposition fluxes of gaseous NH_3_, NO_2_ and HNO_3_, and particulate NH_4_^+^ and NO_3_^−^ for 32 monitoring sites. Additional information such as site code, site name, site coordinate (longitude and latitude), land-use type, sampling method, and sampling period associated with each Nr species is included in ‘profile file’ (Fig. 1). All mean values in the ‘data file’ are monthly data. The ‘readme file’ explains the abbreviations used in the ‘data file’ and ‘profile file’, and provides the units of all variables included (Fig. 1).

### Data acquisition

Ambient concentrations of gaseous NH_3_ and HNO_3_, and particulate NH_4_^+^-N and NO_3_^−^-N, were measured using an active DELTA (DEnuder for Long-Term Atmospheric sampling) system^16^. The system consists of a denuder filter sampling train, a low-volume pump to provide sampling flow rate of 0.2–0.4 L min^−1^, and high sensitivity dry gas meter to record sampled volume. The sampling train comprises of two potassium carbonate plus glycerol (1% (m/v) K_2_CO_3_ + 1% (m/v) glycerol in methanol) coated denuders in series to collect acid gases (HNO_3_, SO_2_ and HCl), followed by two citric acid (5% (m/v) citric acid in methanol) coated denuders to trap NH_3_. Two set of coated cellulose filters in a 2-stage filter pack at the end of the sampling train was used to collect aerosol components, with a first K_2_CO_3_/glycerol impregnated filter to capture particle phase anions (NO_3_^−^, SO_4_^2−^, Cl^−^) and cations (Na^+^, Mg^2+^, Ca^2+^), and a second filter coated with citric acid to collect any volatilized aerosol NH_4_^+^ (Fig. 1). With a monthly sampling period, the detection limits of the DELTA method for gaseous HNO_3_ and NH_3_, and particulate NH_4_^+^ and NO_3_^−^ was determined as 0.03 μg HNO_3_ m^−3^, 0.01 μg NH_3_ m^−3^, 0.02 μg NH_4_^+^ m^−3^ and 0.05 μg NO_3_^−^ m^−3^, respectively.

Gaseous NO_2_ concentration was measured in triplicate with passive Gradko diffusion tubes (Gradko International Limited, UK). Each sampler is made up of acrylic tube (71.0-mm long × 11.0-mm internal diameter) with colored and white thermoplastic rubber caps. Gaseous NO_2_ was absorbed into a 20% triethanolamine/deionized water solution coated onto two stainless steel wire meshes within the colored cap. As indicated by the manufacturer, the uptake rate of the tube is 68.8 × 10^−6^ m^−3^ h^−1^, the desorption efficiency is 0.98, the detection limit is 1.6 μg NO_2_ m^−3^ over a 2-week sampling period, and the analytical expanded measurement uncertainty is ±10%. Over the entire period, the standard deviations of three NO_2_ replicates across all sites were between 0.03 and 20.3 μg NO_2_ m^−3^ and averaged 1.68 μg NO_2_ m^−3^ (95% confidence interval 1.58–1.81). The air intakes of the DELTA system and the NO_2_ tubes were set at 2 m above the ground/vegetation at most sites. All sampling was performed on a monthly basis, that is, one sample per month for each Nr species.

Precipitation samples (here termed as wet/bulk deposition, which contains wet and some dry deposition) at all sites were collected using a standard precipitation gauge (SDM6, Tianjin Weather Equipment Inc., China) located beside the DELTA system (∼2 m). The precipitation gauge consists of a stainless steel funnel and glass bottle (vol. 2000–2500 ml). Precipitation amount was measured using a graduated cylinder (scale range: 0–10 mm; division: 0.1 mm) coupled with the gauge. After each daily (8:00 am–8:00 am next day) event, precipitation samples (including rain and melted snow) were collected and stored in clean polyethylene bottles (50 mL) at −18 °C until delivery to the laboratory at China Agricultural University (CAU) for chemical analysis. The collectors were cleaned three times with high-purity water after each collection and once every week in order to avoid cross contamination.

In the CAU’s analytical laboratory, the exposed sampling trains of the DELTA systems and NO_2_ tubes were stored at 4 °C before analysis. Acid-coated denuders and aerosol filters were extracted with 6 and 10 mL of high-purity water (18.2 MΩ), respectively, and analyzed for NH_4_^+^-N with an AA3 continuous-flow analyzer (CFA) (BranC Luebbe GmbH, Norderstedt, Germany). Carbonate-coated denuders and filters were both extracted with 10 mL 0.05% H_2_O_2_ solution followed by analysis of NO_3_^+^-N using the same CFA. The detection limits were determined as 0.01 mg N L^−1^ for NH_4_^+^ and NO_3_^−^. The meshes from the NO_2_ diffusion tubes were extracted with a solution containing sulfanilamide, H_3_PO_4_, and N-1-naphthylethylene-diamine, and the NO_2_ content in the extract determined using a colorimetric method by absorption at a wavelength of 542 nm^9^. The detection limit for NO_2_ was 0.01 mg N L^−1^. Each collected precipitation sample was filtered with a 0.45 µm syringe filter (Tengda Inc., Tianjin, China), and analyzed for NH_4_^+^-N and NO_3_^−^-N using the CFA as mentioned above. Quality assurance and quality control procedures adopted in the analytical laboratory are described in “Technical Validation”. Further details of precipitation measurement, samples handling, and chemical analysis can be found in our previous studies^9,11^.

Wet/bulk N deposition flux *(D*_*w*_, kg N ha^−1^) was calculated as the product of the precipitation amount (*P*_*t*_, mm) and the volume-weighted mean concentration of Nr species in precipitation (*C*_*w*_, mg N L^−1^), using Equation (1).

1 $${D}_{w}={P}_{t}{C}_{w}/100$$

The dry deposition flux of gaseous and particulate Nr species was calculated by multiplying measured concentrations with simulated deposition velocities (*V*_*d*_) from the GEOS (Goddard Earth Observing System)-Chem chemical transport model (http://geos-chem.org)^17^. The GEOS-Chem CTM is driven by GEOS (Goddard Earth Observing System) assimilated meteorological data from the NASA Global Modeling and Assimilation Office (GMAO). The GEOS-5 data are available with a temporal resolution of 6 h (3 h for surface variables and mixing depths) and a horizontal resolution of 1/2° latitude × 2/3° longitude. The nested-grid version of GEOS-Chem^18^ was used with the native 1/2° × 2/3° resolution over East Asia (70°E–150°E, 11°S–55°N). The model calculation of dry deposition of Nr species follows a standard big-leaf resistance-in-series model for gases^19^ and aerosol^20^. The aerodynamic resistance to turbulent transfer from the measurement heights (~2 m) to the roughness height is estimated using the GEOS-5 data. The surface uptake resistance is calculated based on the Global Land Cover Characteristics Data Base Version 2.0 (http://edc2.usgs.gov/glcc/globdoc2_0.php), which defines land types (e.g., urban, forest, etc.) at 1 km × 1 km resolution and is then binned to the model resolution as fraction of the grid cell covered by each land type. The model 1/2° resolution thus coarsely represents regional land characteristics around the monitoring sites. Bi-directional NH_3_ exchange is not considered in the model. The hourly *V*_*d*_ values were modeled from January 2011 to May 2013, and the period of June 2013-December (when the GEOS-5 meteorological data are unavailable) was filled using mean modeled values for each hour. The monthly *V*_*d*_ at each site was then averaged based on the hourly dataset for further estimation of dry deposition flux of each N_r_ species during the observation.

## Data Records

The data are available in a single dataset^21^, which consists of three Microsoft Excel files: the ‘data file’ (NNDMN 1.0 Data File), the ‘profile file’ (NNDMN 1.0 Profile File), and the ‘readme file’ (NNDMN 1.0 Read Me) which explains the abbreviations and units (Fig. 1). The NNDMN 1.0 database is the most comprehensive and up-to-date measurement-based dataset of ground-level concentrations and dry and bulk deposition of key Nr species over different land-use types (e.g., urban, rural, and coastal, forest, grassland) in China. The data time series runs from 2010 to the current latest available year of 2015. Specifically, the NNDMN 1.0 database includes records at 32 locations for monthly mean concentrations and bulk deposition of fluxes of NH_4_^+^ and NO_3_^−^ in precipitation, and monthly mean air concentrations and dry deposition fluxes of NH_3_, NO_2_, HNO_3_, and particulate NH_4_^+^ and NO_3_^−^, with summary statistics presented in Table 1. In brief, monthly mean concentrations of NH_3_, NO_2_, HNO_3_, and particulate NH_4_^+^ and NO_3_^−^ ranged over 0.16–39.6 (average 7.0), 0.13–29.1 (6.6), 0.02–4.9 (1.2), 0.02–57.2 (6.5), and 0.01–32.1 μg N m^−3^ (2.8 μg N m^−3^), respectively, while monthly volume-weighted mean concentration of NH_4_^+^ and NO_3_^−^ in precipitation were 0.01–26.8 (2.3), and 0.02–28.9 mg N L^−1^ (2.4 mg N L^−1^), respectively. The averages of matched months during the sampling period at each site were used to calculate annual averages. Grouped by land-use type, the annual average dry deposition (1.9–16.6 kg N ha^−1^ yr^−1^ for NH_3_, 0.2–16.0 kg N ha^−1^ yr^−1^ for HNO_3_, 0.2–9.5 kg N ha^−1^ yr^−1^ for NO_2_, 0.1–11.8 kg N ha^−1^ yr^−1^ for particulate NH_4_^+^, and 0.2–4.1 kg N ha^−1^ yr^−1^ for particulate NO_3_^−^) and bulk deposition (2.7–18.9 kg N ha^−1^ yr^−1^ for NH_4_^+^-N, and 1.5–17.4 kg N ha^−1^ yr^−1^ for NO_3_^−^-N) of inorganic Nr species are ranked by land use as urban > rural > background sites (the latter comprising the average of forest, grassland and coastal sites). In addition, across all sites the total dry deposition for the five Nr species (21.9 ± 10.8 kg N ha^−1^ yr^−1^ (mean ± standard deviation)) was similar to the total bulk deposition of NH_4_^+^-N and NO_3_^−^-N (21.5 ± 6.9 kg N ha^−1^ yr^−1^). These results reflect the positive association between anthropogenic N emissions and N deposition, and also demonstrate that dry deposition is a major pathway in China that must be included in estimates of the total Nr deposition.

**Table 1:** Summary statistics for monthly mean concentrations of Nr species in air (NH_3_, NO_2_, HNO_3_, pNH_4_^+^ and pNO_3_^−^ in μg N m^−3^) and in precipitation (NH_4_^+^ and NO_3_^−^ in mg N L^−1^), and their respective dry and bulk deposition fluxes (kg N ha^−1^ month^−1^) during the sampling period at the 32 sites.

Species^a^	Concentration						Deposition flux					
	Min	Max	Median	Mean	SD	N	Min	Max	Median	Mean	SD	N^b^
NH_3_	0.16	39.6	5.8	7.0	5.4	1790	0.01	4.33	0.63	0.80	0.66	1790
NO_2_	0.13	29.1	5.8	6.6	3.8	1790	0.0002	2.38	0.19	0.26	0.26	1790
HNO_3_	0.02	4.9	1.0	1.2	0.7	1790	0.0004	3.88	0.29	0.43	0.46	1790
pNH_4_^+^	0.02	57.2	4.9	6.5	6.1	1790	0.0013	4.64	0.19	0.31	0.36	1790
pNO_3_^−^	0.01	32.1	2.1	2.8	2.5	1790	0.0002	1.37	0.09	0.12	0.11	1790
NH_4_^+^	0.01	26.8	1.4	2.3	2.7	1426	0	10.4	0.55	0.95	1.24	1619
NO_3_^−^	0.02	28.9	1.5	2.4	2.9	1426	0	10.7	0.57	0.86	1.03	1619

^a^pNH_4_^+^ and pNO_3_^−^ denote particulate NH_4_^+^ and NO_3_^−^; NH_4_^+^ and NO_3_^−^ denote NH_4_^+^ and NO_3_^−^ in precipitation.

^b^The number of 1619 included value of 0 from sampling months without precipitation event.

## Technical Validation

For all sites in the NNDMN, the data were obtained via internationally widely-used samplers (e.g., rain gauge, DELTA system and Gradko tubes) and quantification methods (e.g., inferential method). In addition, all field measurements were subject to uniform standard procedures for sampling and storage methods, undertaken by trained personnel. All samples were prepared and measured in the Key Laboratory of Plant-Soil Interactions, Chinese Ministry of Education, China Agricultural University, which operates a full formal quality control system^22^. Three laboratory blanks and three field (travel) blanks were prepared and analyzed for each batch of field exposed samples. The laboratory blanks were subtracted from the samples to correct all quantified species concentrations, and field (travel) blanks were used to check for contamination. Quality assurance was routinely (once every 20 samples) performed using standard (designed specific concentrations of NH_4_^+^-N and NO_3_^−^-N) samples during each analysis run. The differences between the determined and “theoretical” results from the standard samples were controlled to be less than ±5%. In addition, triplicate NO_2_ samplers were deployed and collected at all sites for each sampling, which allowed confirming a good reproducibility of the Gradko method. The NNDMN 1.0 database presents detailed information on the sampling and laboratory analysis methods for the users to evaluate for themselves. Where there was occasional missing monthly data, the averages of the previous and following months were used to gap fill for calculation of the monthly mean (which have been highlighted in red in the ‘data file’, and were also specified in ‘readme file’). Data from this NNDMN 1.0 dataset has been subject to international peer review as part of the process of publication in high-quality international literature^9,11^.

### Ancillary datasets

Other datasets that users may find helpful in interpreting the data provided in the NNDMN 1.0 database described in this paper include the following.

- The MIX gridded emissions inventory for anthropogenic pollutants (e.g., NH_3_ and NO_*x*_) and greenhouse gases (http://meicmodel.org/)
- The Infrared Atmospheric Sounding Interferometer (IASI) NH_3_ data (https://iasi.aeris-data.fr/NH3/)
- The Ozone Monitoring Instrument (OMI) NO_2_ data (http://www.temis.nl/airpollution/NO2.html)
- Precipitation and other meteorological data for China (http://data.cma.cn/en)
- Land-use cover for China (http://www.resdc.cn/)
- Air quality data for China (http://www.cnemc.cn/).

## ISA-Tab metadata file

Download metadata file

## Data Availability

No custom computer code was used to generate the data described in the manuscript.
